# Entrepreneurial Leadership and Entrepreneurial Performance in Start-Ups: A Moderated Serial Mediation Model

**DOI:** 10.3389/fpsyg.2022.831555

**Published:** 2022-02-18

**Authors:** Bo Pu, Siyu Ji, Wenyuan Sang, Zhiwei Tang

**Affiliations:** ^1^School of Public Affairs and Administration, University of Electronic Science and Technology of China, Chengdu, China; ^2^School of Business and Tourism, Sichuan Agricultural University, Chengdu, China

**Keywords:** entrepreneurial leadership, tacit knowledge sharing, job embeddedness, career growth opportunities, entrepreneurial performance

## Abstract

The purpose of this study is to explore the impact of entrepreneurial leadership on entrepreneurial performance in start-ups. Specifically, a moderated serial mediation model was developed to investigate the mediating role of tacit knowledge sharing and job embeddedness and the moderating effect of career growth opportunities. Data was collected from 376 start-up employees *via* an online survey platform. Using hierarchical multiple regression and Hayes' PROCESS Macro by SPSS 21.0, and structural equation modeling by AMOS 23.0, support was found for both mediation and moderation effects. Results showed that entrepreneurial leadership significantly positively affects entrepreneurial performance by mediating with tacit knowledge sharing and job embeddedness. Moreover, career growth opportunities moderate the serial mediating effect of tacit knowledge sharing and job embeddedness between entrepreneurial leadership and entrepreneurial performance. This study provides theoretical guidance for entrepreneurial leadership to improve entrepreneurial performance.

## Introduction

The rapid development of the Internet has given rise to a boom in entrepreneurship. According to the Global Entrepreneurship Monitor's 2018/2019 global report, “79.3% of respondents think entrepreneurship is a good career choice” (Bosma and Kelley, 2019). However, not all start-ups end up achieving success. Many entrepreneurs who begin their ventures with great enthusiasm are eventually eliminated from the market as a result of not achieving satisfactory business performance. About 700,000 new enterprises are born in the United States every year, of which only 3.5% become large enterprises (Barringer et al., 2005). The iteration of information technology has made the entrepreneurial environment even more complex. How can leaders reorganize and lead their organizations and employees to move forward in this increasingly uncertain environment? How can the process of starting a business be made more rewarding? How can leaders retain employees in a rapidly changing competitive landscape with increasing risks? How might leaders motivate employees to perform well? These are the questions that start-ups face, and the questions addressed by this study. In extant leadership research, entrepreneurial leadership (EL) has not been fully explored in-depth; as such, this study seeks to clarify the effect of EL on entrepreneurial performance (EP) in start-ups.

EL is often characterized by both industries and academia as a high-performance leadership style that actively promotes trust and teamwork. EL emphasizes innovation, actively drives employees to create new ideas, and involves a willingness to take on risky projects (Sayyam et al., 2022). EL is extremely beneficial for start-ups, as an effective entrepreneurial leader can provide key resources and information to gain support from key stakeholders, allowing for business development (Dabić et al., 2021). Key characteristics of an entrepreneurial leader are adeptness at seeking and developing entrepreneurial opportunities, active coordination and planning (Renko et al., 2015), and an emphasis on adaptive and innovative actions (Gupta et al., 2004). When considering the significance of leaders to enterprises, scholars have mainly discussed leaders' level of experience and charisma (Eesley and Roberts, 2012), paying less attention to the processes by which leadership influences behavioral and psychological changes in employees' work. Therefore, this study focuses on analyzing the process by which EL impacts employees' work behavior and psychology. In explaining the role of EL, existing studies have mainly discussed the consequences of EL at the individual, team, and organization levels. Specifically, EL has a positive impact on organization development (Mehmood et al., 2021), firm growth (Koryak et al., 2015), and employee innovative behavior (Akbari et al., 2020). EL is closely related to EP, but further analysis is needed to determine the specific paths involved (Nguyen et al., 2021). Paudel (2019) explored the mediating mechanism between EL and performance at the organizational level, and noted the mediating role of organizational innovation. A recent study analyzed the mediating role of innovation capability between EL and performance, using Indonesian SMEs as respondents (Purwati et al., 2021). In practice, the factors that influence EP do not just occur at a single level, but are the result of a combination of multiple influences, such as individuals and team organizations. However, most studies have been monolithic, considering only leaders or groups. Therefore, this study analyzes the impact of EL on EP at both the individual and organizational level. The goal is to analyze and disclose the impact of EL on EP in a comprehensive and in-depth manner.

The mediating mechanism discussed in this study is inspired by the resource-based view. Resource-based view argues that firms generate a competitive advantage from their unique combination of resources (Ferreira et al., 2011). Performance is inseparable from the efforts of managers and employees (Nguyen et al., 2021). Undoubtedly, knowledge and talent are two key resources, and EL cannot function without them. In order to address the aforementioned gaps in the extant research regarding the impact of EL on EP, this study introduces two mediating variables: tacit knowledge sharing (TKS) at the team level and job embeddedness (JE) at the individual level.

Scholars have two main approaches to describe EL: one defines it as work-oriented, and the other explains it as socio-cultural and contextual (Bagheri and Pihie, 2011). EL and its related concepts are understood in Western contexts, but they are not well studied in the East and developing countries (Paudel, 2019). One study analyzed the feasibility of EL in China based on exploratory cases (Wang et al., 2012). However, the mechanism of EL's influence on EP has not been carefully examined. The context of EL in China is dynamic and complex, and the interaction between philosophical traditions and cultural values cannot be ignored. Therefore, it is necessary to examine and analyze the influence of EL on EP in the Chinese context. EL actively promotes sharing and discussion among employees so that they may take initiative to help others. After experiencing this environment of generosity, employees are compelled to remain with the organization or the team. The reciprocity principle of social exchange theory (SET) plays a vital role in explaining the internal mechanism of the relationship between leadership style and subordinate behavior in Chinese enterprises (Chen et al., 2014). According to SET, people strive to make their social interactions maximally beneficial to themselves. The SET literature suggests that exchanges at work can occur in the context of seven resources, namely goods, services, money, love/emotion, status, information, and work itself (Cox, 1999). Therefore, this study first examines how job development is pursued by employees in start-ups, then introduces career growth opportunities (CGO) to explore the moderating effect on the mechanism by which EL influences EP.

The purpose of this study is to explore the mechanisms of influence between EL and EP, how EL affects employee psychology and behavior, and how EL functions to retain employee loyalty. This study will enable leaders to understand how to motivate their employees and improve business performance, and allow them to adopt appropriate incentives to promote beneficial employee behavior.

## Literature Review and Hypotheses

### The Direct Influence of EL on EP

The entrepreneurial environment of start-ups is chaotic and the responsibilities of team members are often unclear (Freeman and Siegfried Jr, 2015). Leaders must incentivize their employees to make the entire organization run in an orderly manner. EL plays the role of a “guiding light” for start-ups, providing support and vision to team members (Freeman and Siegfried Jr, 2015). The entrepreneurial leader has excellent job skills and personal charisma to accomplish the specific tasks and responsibilities of the start-up. Employees can establish achievable and innovative goals and strive for them with the guidance of such a leader. An entrepreneurial leader is also adept at seizing entrepreneurial opportunities and avoiding risks to achieve entrepreneurial goals by influencing the actions of their followers (Musara and Nieuwenhuizen, 2020). EL can greatly motivate employees and develop organizational commitment (Cai et al., 2019). Organizational development requires the continuous efforts of the entrepreneurial leader and their employees, as people in an organization are the drivers of creativity (Tóth et al., 2020).

EP is the extent to which a firm achieves a specific task or goal during the entrepreneurial process and is a key factor in explaining entrepreneurial outcomes and the enterprise's overall competitive advantage. EP can be interpreted as entrepreneurial willingness, the ability to identify opportunities, or business success (Baron, 2004). One study examined the impact of the interaction between EL and the creativity of entrepreneurial team members on the innovative capacity of new firms using patent creation as a measure (Chen, 2007). Previous studies point to the positive impact of entrepreneurial orientation on EP (Rauch et al., 2009), and the effect of authentic leadership on EP has been confirmed (Shirey, 2006). In an unpredictable environment, a company's survival depends on managers' entrepreneurial spirit and leadership (Demartini and Beretta, 2019). EL is particularly important in the start-up phase of a new business, focusing on empowering employees, which helps to enhance their self-efficacy and entrepreneurial abilities (Guberina and Wang, 2021). EL will motivate employees to be more proactive in pursuing organizational goals, thus improving organizational performance and value creation (Alsharif et al., 2021). Therefore, this study posits that there is a positive correlation between EL and EP, hypothesized as follows.

*H1:* EL is positively associated with EP in start-ups.

### Mediating Role of TKS and JE

An organization's ability to innovate and sustain a competitive advantage is inextricably linked to knowledge sharing (Terhorst et al., 2018). Knowledge sharing among individuals is the key to creating value, gaining a competitive advantage, and improving overall organizational performance (Obrenovic et al., 2020). Knowledge sharing can be classified as either explicit and tacit (Zhao et al., 2020). Tacit knowledge refers to the skills accumulated by employees during their tenure and is abstract expertise (Obrenovic et al., 2021) which facilitates the development of employees and companies. Tacit knowledge is highly personalized, based on the accumulation of personal experience, and influenced by personal subjective factors (Wang et al., 2006). The cost of building tacit knowledge for individuals is high (Kogut and Zander, 1992), so employees need motivation to share tacit knowledge. Leadership can motivate employees to actively provide and share their ideas (Edú-Valsania et al., 2016). A study on small craft enterprises in Bali demonstrated a positive association between EL and knowledge sharing (Riana et al., 2020). EL is characterized by flexibility, patience, persistence, adventure, tenacity, self-confidence, motivation, and initiative (Renko et al., 2015), improving employees' trust in their team and leaders and making them more willing to share tacit knowledge. Knowledge sharing contributes to innovation (Fernandes Crespo et al., 2021), and is a key part of knowledge management; this helps organizations achieve a sustainable competitive advantage (Han et al., 2020), and contributes positively to organizational performance (Orpipath, 2018). Leaders can positively contribute to employee tacit knowledge sharing (TKS) by providing incentives and empowerment (Obrenovic et al., 2021). A recent study explored the role of tacit knowledge in driving business model innovation among team members and team leaders through an Italian engineering consulting firm (Castellani et al., 2021). Therefore, this study argues that TKS mediates the positive influence of EL on EP. The following hypothesis is proposed.

*H2:* The positive association of EL on EP is mediated by TKS.

Job embeddedness (JE) refers to the combination of forces that prevent individuals from leaving their jobs (Mitchell et al., 2001). Specifically, it includes a set of social, economic, and psychological factors that influence individuals to stay in their jobs (Kiazad et al., 2015). JE is important in organizational settings because it refers to the factors which motivate employees to continue working (Karatepe, 2013) and to stay in their current organizations. JE is often used to explain employees' turnover intention (Mitchell et al., 2001). Scholars have found that JE can also be used to predict other job-related results (Ghosh et al., 2017).

Organizational leadership has been proven to positively impact employees' JE (Holmes et al., 2013). JE is considered a beneficial state to both the organization and the individual. Employees with high JE tend to have higher work ethic, superior productivity, and excellent innovative behavior (Norouzinik et al., 2021). According to JE theory, a meaningful connection and good fit between the employee, the organization, and the job will motivate good performance (Lee et al., 2004). JE reflects the relationship between leaders and followers, and the behavioral characteristics of leaders influence employees' JE (Mitchell et al., 2001). High JE employees go the extra mile to stay in the organization by performing better and achieving their personal career goals (Al-Ghazali, 2020). They develop more positive qualities and create value for the organization (Norouzinik et al., 2021). JE mediates between job factors and key organizational results (Holtom et al., 2006). Therefore, it is believed that JE mediates between EL and EP.

*H3:* The positive association of EL on EP is mediated by JE.

The more connected people are to others, their workplace, and their community, the less likely it is that they will leave their jobs (Lee et al., 2004). Employees improve their JE when they feel socially connected and when their skills and knowledge are enhanced (Karatepe, 2013). When employees are in an atmosphere where they are willing to share tacit knowledge, they will inevitably strengthen their attachment to the organization and will not leave it. For knowledge sharers, when the organization or their colleagues recognize the knowledge they contribute, it enhances their reputation, gains them respect, and makes them more enthusiastic about their work. As was mentioned earlier, information exchange is a type of social exchange. When employees feel that the tacit knowledge shared by their colleagues is beneficial to them, they have a reason to stay in the organization. In return, they will contribute more to the organization. This study investigates a link between TKS and JE when mediating EL and EP, and proposes the following hypothesis.

*H4:* TKS and JE act as serial mediating factors in the positive relationship between EL and EP.

### Moderating Role of CGO

Career growth opportunities (CGO) are opportunities provided by managers for employees to enhance their job competence and expertise, allowing the employees more room for development and responsibility at work. They are a kind of stimulation and motivation for employees (Weer and Greenhaus, 2020). Employees can keenly perceive the CGO of their organizations (Weng and McElroy, 2012). Career-focused employees are more likely to pursue performance and develop a desire for advancement than less career-focused employees. Leaders or internal incentives strongly influence these employees, and corporate leaders should strive to take advantage of such strong motivation (Weer and Greenhaus, 2020). From the perspective of self-determination theory (Deci et al., 1994), CGO is an informational environmental factor. When employees acquire or perceive better CGO, they are more enthusiastic about their work, more comfortable with their work, and capable of controlling their work resources. Specifically, they are actively engaged, energetic, and committed at work (Schaufeli and Bakker, 2004).

In general, the more CGO employees perceive in the organization, the higher the organization's care and support for the employee (Lu et al., 2016). The occurrence of employees' TKS comes from a combination of personal and external factors. The factors that influence unique behaviors in a given situation result from the interaction of individual characteristics and external conditions. Personal factors are generally inherent and unshakable, but external factors with motivational effects have room for improvement. Incentive policies offered by organizations can effectively promote knowledge sharing (Orpipath, 2018). CGO provides a unique environment for organizations that may influence the effectiveness of leaders through the attitudes and behaviors of subordinates (Bashir et al., 2020). When employees perceive that the organization provides more CGO, they develop positive work emotions and form psychological attachments (Wang et al., 2014). A Chinese proverb says, “A drop of water should be repaid with a spring.” When employees are aware of the “water” provided by the organization, they will naturally be more willing to reciprocate and are more inclined to share tacit knowledge with their colleagues, thus promoting their sense of belonging to their jobs and improving their performance. This is also reflected in the reciprocity principle of SET: when individuals feel support from the organization, they have a stronger sense of belonging and a deeper sense of identity. Furthermore, when employees feel motivated by the organization's supportive measures, an employee will gladly perform TKS to promote the organization's progress. Employees will feel obligated to remain loyal to the company (Bashir et al., 2020) and stay embedded in the organization. Subsequent research reaffirms this (Lu et al., 2016), and additionally exemplifies the “links” in JE theory (Mitchell et al., 2001). Bashir et al. (2020) confirmed that CGO moderates the relationship between procedural justice and organizational commitment. Therefore, this study makes the following hypotheses.

*H5:* CGO moderate the positive relationship between EL and TKS; the higher the CGO, the more significant the positive influence of EL on TKS.
*H6:* CGO moderate the mediating association of TKS between EL and TKS; the higher the CGO, the more significant the mediating association of TKS between EL and EP.
*H7:* CGO moderate the serial mediating association of TKS and JE between EL and TKS; the higher the CGO, the more significant the serial mediating association of TKS and JE between EL and EP.

**Figure 1:**
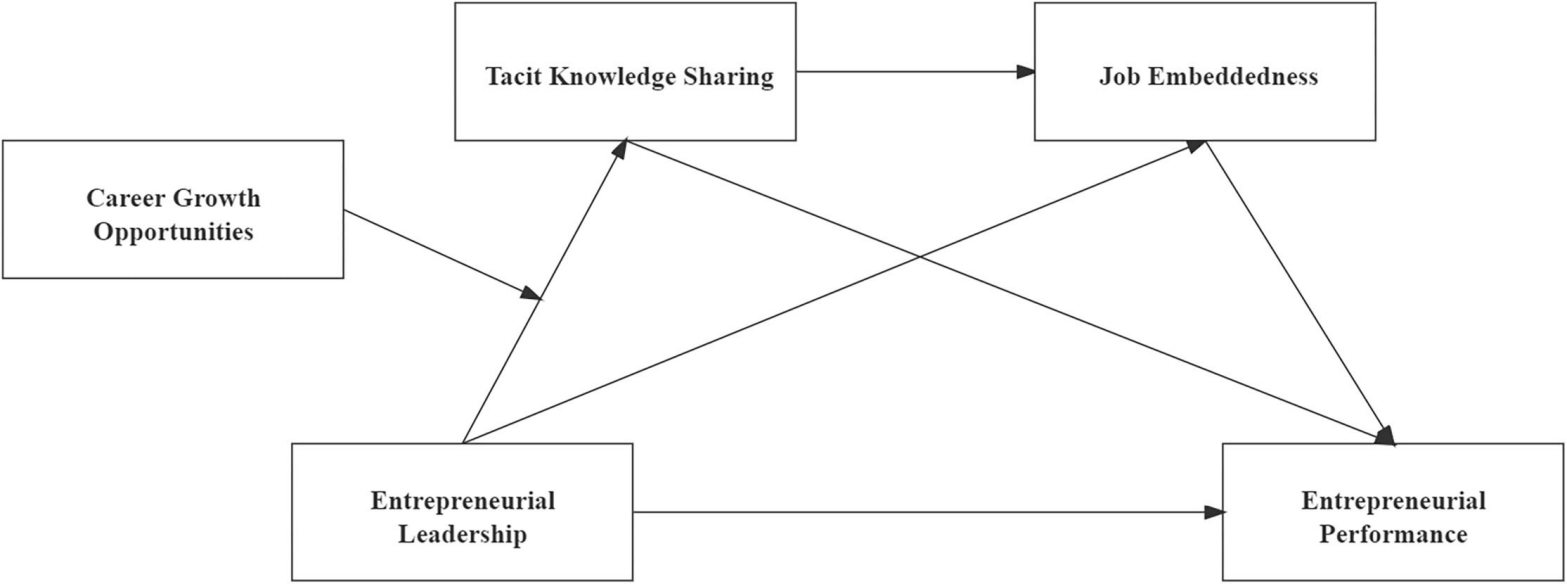
Conceptual model.

To sum up, our conceptual model is shown in Figure 1.

## Methods

### Sample and Procedures

The survey data was collected through the online survey website “Wenjuanxing”. Participants were employees of Chinese start-ups established in the past 5 years. Several efforts were made to reduce common method variance. Firstly, to weaken the chance of comparison among respondents and improve the data quality of this study, the data of no more than ten employees per company was collected. Secondly, before data collection, participants were informed that filling out the questionnaire is anonymous and that the information they provide will be used for research only. Thirdly, Internet protocol access was restricted to avoid repeated surveys. Employees were asked about their experiences with EL and their shared work experiences, including TKS, JE, CGO and EP. This survey was closed after collecting 500 responses. After excluding questionnaires with responses that were too long or too short and contradictory answers, 376 valid questionnaires were obtained, representing a validity rate of 75.2%. Of these, 136 respondents (36.2%) were male, and 240 (63.8%) were female. Respondents were mainly between 21 and 40 years old (96.5%). The majority of respondents had a bachelor's degree or higher (79.5%), and most respondents had a monthly salary of between ¥3,000 and ¥8,000 (60.9%). Respondents came from various companies, most of which had fewer than 100 employees (75.5%).

### Measures

The maturity scales published in national and international journals were used in this study. All English scales were translated using a standard translation back-translation procedure to avoid semantic ambiguity to the greatest extent possible. Second, given the sensitivity and covert nature of corporate surveys, all variables in this study were appropriate for use in an informal setting and were measured by anonymous self-assessment. All items were assessed using Likert 7-point scales, ranging from 1 (strongly disagree) to 7 (strongly agree).

#### EL

Because the EL scale developed by Gupta et al. (2004) had many items, an item parceling method (Matsunaga, 2008) was used to parcel five indicators based on the five dimensions of EL. The five dimensions are building challenges, absorbing uncertainty, clearing the way, building commitments, and specifying constraints. In this study, Cronbach's alpha for the five-item scale was 0.870.

#### JE

JE was measured using the seven-item scale developed by Crossley et al. (2007). A reliability analysis was conducted during the pre-study, and two items were found not to meet the requirements and were removed. In this study, Cronbach's alpha for the five-item scale was 0.845.

#### TKS

TKS was measured using a three-item scale from Lee's (2001) study on TKS. In this study, Cronbach's alpha for the three-item scale was 0.792.

#### CGO

In this study, the scale used by Van Veldhoven and Dorenbosch (2008) was selected to measure the CGO experienced by employees at work, with appropriate modifications being made to the items so they were suitable for the present study. Cronbach's alpha for the three-item scale was 0.800.

#### EP

A four-item EP scale was create based on the classic scale of Chandler and Hanks (1993), with appropriate modifications being made to meet the needs of this paper. In this study, Cronbach's alpha for the four-item scale was 0.789.

#### Control Variables

Scholars have noted that the age and salary of employees, the number of employees at a company, and company tenure have a significant impact on EP (Eesley and Roberts, 2012). Therefore, we controlled for employees' demographic characteristics and structural variables.

### Data Analysis

The data analysis process for this study was as follows. First, validation factor analysis was performed using Amos 23.0 to test the discriminant validity between EL, TKS, JE, CGO, and EP. Second, the relationship between the variables was analyzed using SPSS 21.0. Third, hierarchical multiple regression analysis (Baron and Kenny, 1986), structural equation modeling, and SPSS PROCESS Macro (Hayes, 2013) were used to test the hypotheses.

## Results

### Confirmatory Factor Analysis

Because the EL, TKS, JE, CGO, and EP data come from the same source, the possibility of a common method variance existing between the five constructs could not be disregarded. Firstly, the Harman single factor method was used to test common method variance (Podsakoff et al., 2003). After unrotated exploratory factor analysis for all items of the study variables, the total variance explained by all factors with characteristic roots greater than 1 was 59.6%. The various explanation of the first principal component was 30.6%. The variance explained by this factor did not exceed 40%. There is no serious common method variance problem in which a single factor explained most of the variance of all variables. In addition, to estimate the homoscedasticity bias and measure the discriminant validity of the variables, a confirmatory factor analysis was performed using Amos 23.0 (Table 1). Based on the results of the validation factor analysis and the corrected indicators for each indicator variable, some items were removed so that all questionnaire items had factor loadings greater than 0.5 (Hair et al., 2014). According to the standard for model fit: χ ^2^*/df* < 3 (Kline, 2015); GFI > 0.80 (Doll et al., 1994); AGFI > 0.80 (MacCallum and Hong, 1997); RMSEA < 0.08 (Hu and Bentler, 1999); TLI > 0.90 (Hu and Bentler, 1999); CFI > 0.900. Table 1 shows that the five-factor model outperformed the other models (χ ^2^*/df* = 1.757; GFI = 0.931; AGFI = 0.909; CFI = 0.966; RMSEA = 0.025; TLI = 0.960).

**Table 1:** Confirmatory factor analysis results.

**Model**	**Factor**	***χ^2^***	***df***	***χ^2^/df***	**GFI**	**AGFI**	**CFI**	**RMSEA**	**TLI**
Single-factor	EL+TKS+CGO+JE + EP	922.848	170	5.429	0.768	0.713	0.790	0.109	0.765
Two-factor	EL, TKS+CGO + JE + EP	701.282	169	4.150	0.818	0.774	0.851	0.092	0.833
Three-factor	EL, TKS+CGO + JE, EP	555.931	167	3.329	0.850	0.811	0.891	0.079	0.876
Four-factor	EL, TKS+CGO, JE, EP	381.991	164	2.329	0.904	0.877	0.939	0.060	0.930
Five-factor	EL, TKS, JE, EP, CGO	281.076	160	1.757	0.931	0.909	0.966	0.025	0.960

*n = 376*.

### Descriptive Statistics

Table 2 shows the mean, standard deviation, correlation coefficient, average variance extracted (AVE), and composite reliability (CR) of all variables involved in this study. There is a significant positive correlation between EL and TKS (*r* = 0.541, *p* < 0.001), JE (*r* = 0.569, *p* < 0.001), CGO (*r* = 0.587, *p* < 0.001), and EP (*r* = 0.606, *p* < 0.001). The results of variable correlation analysis preliminarily verify the research hypotheses of this paper. The AVE from all variables (except EP) is >0.5, which is an acceptable value (Fornell and Larcker, 1981), and the CR is >0.7 (Fornell and Larcker, 1981) (Table 2). Although the AVE of EP is 0.495, its CR is 0.796. The convergence validity of this variable meets the requirements (Fornell and Larcker, 1981).

**Table 2:** Correlation and descriptive statistics.

**Variable**	**Gender**	**Age**	**Monthly salary**	**Number of employee**	**Corporate tenure**	**EL**	**TKS**	**JE**	**EP**	**CGO**
Gender										
Age	−0.025									
Monthly salary	−0.191^***^	0.297^***^								
Number of employee	−0.032	0.195^***^	0.238^***^							
Corporate tenure	0.000	0.398^***^	0.309^***^	0.347^***^						
EL	−0.021	0.092	0.195^***^	0.159^**^	0.237^***^					
TKS	0.088	0.031	0.125^*^	0.184^***^	0.232^***^	0.541^***^				
JE	−0.070	0.211^***^	0.288^***^	0.210^***^	0.380^***^	0.569^***^	0.496^***^			
EP	0.003	0.103^*^	0.165^**^	0.285^***^	0.333^***^	0.606^***^	0.472^***^	0.543^***^		
CGO	−0.035	0.089	0.303^***^	0.292^***^	0.330^***^	0.587^***^	0.585^***^	0.603^***^	0.551^***^	
Mean	0.638	2.282	2.965	2.763	2.713	5.271	5.316	4.812	4.699	5.179
Standard deviation	0.481	1.033	1.005	0.903	1.008	0.696	1.124	1.124	1.101	1.146
AVE						0.576	0.559	0.526	0.495	0.577
CR						0.871	0.791	0.847	0.796	0.803

*Gender: 0 = male, 1 = female; Age: 1 = 22–25 years old, 2 = 26–30 years old, 3 = 31–35 years old, 4 = 36–40 years old, 5 = 41 years old and above; Monthly salary: 1 = ¥3,000 and below, 2 = ¥3,001–¥5,000, 3 = ¥5,001–¥8,000, 4 = ¥8,001–¥15,000, 5 = above ¥15,000; Number of staff: 1 = below 10 persons, 2 = 10-50 persons, 3 = 50–100 persons, 4 = above 100 persons; Corporate Tenure: 1 = below 1 year, 2 = 1–2 years, 3 = 2–3 years, 4 = 3–5 years; n = 376*;

* *p < 0.05 (two-tailed)*,

** *p < 0.01 (two-tailed)*,

*** *p < 0.001 (two-tailed)*.

### Hypothesis Testing

**Figure 2:**
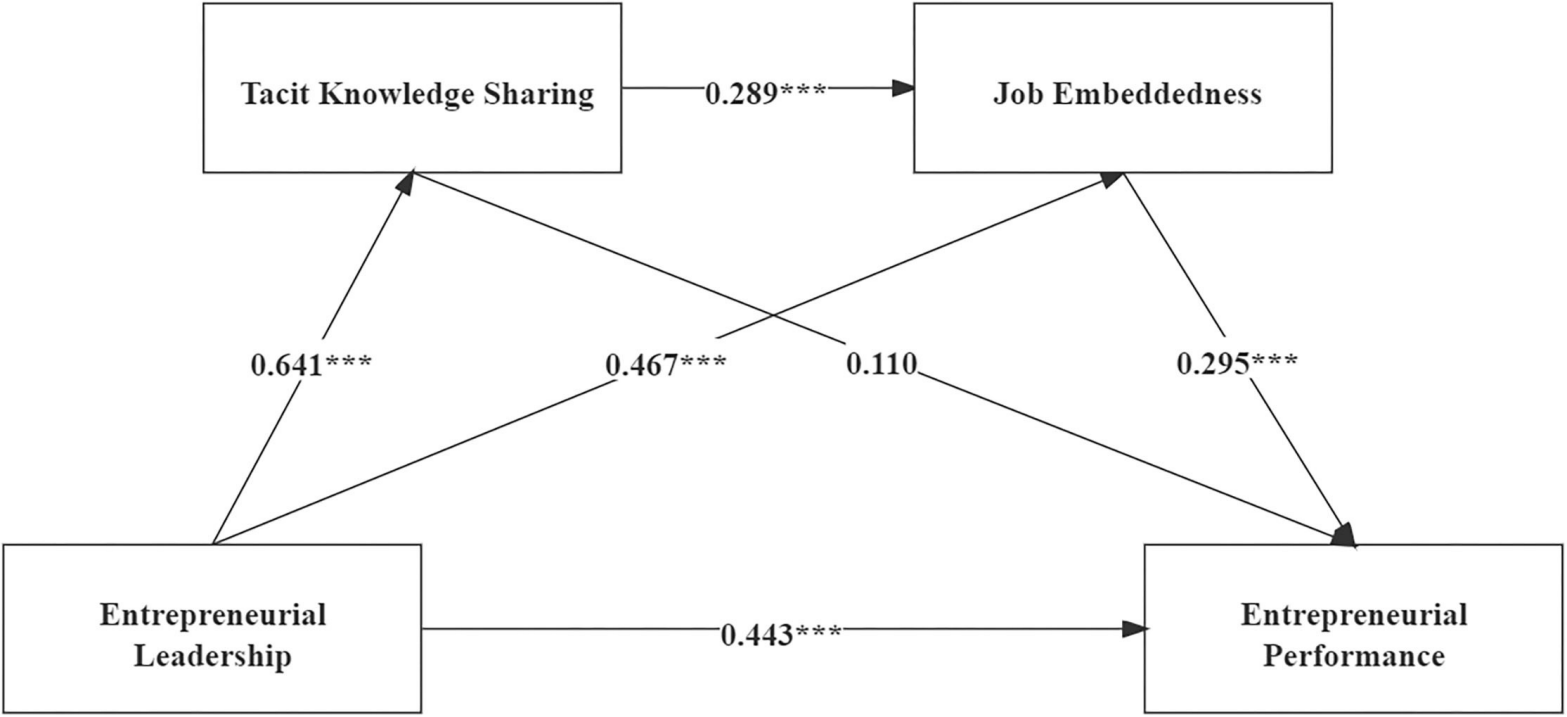
Main effects. *n* = 376; ***p < 0.001.

Following the suggestion of Baron and Kenny (1986), hierarchical regression analysis was applied to test hypothesis 1 (Table 3). The results showed a significant positive association of EL on EP (M6, β = 0.866, *p* < 0.001). Furthermore, a structural equation model was used to test this hypothesis. The main path coefficient (γ) of this model is shown in Figure 2, and EL has a significant positive association on EP (γ = 0.443, *p* < 0.001). Therefore, hypothesis 1 is accepted.

**Table 3:** Hypothesis test results.

	**TKS**		**JE**			**EP**		
	**M1**	**M2**	**M3**	**M4**	**M5**	**M6**	**M7**	**M8**
**Control variables**
Gender	0.240^*^	0.202^*^	−0.089	−0.149	−0.128	0.034	0.028	−0.007
Age	−0.083	−0.044	0.053	0.074	0.094^*^	−0.042	−0.045	−0.033
Monthly salary	0.017	−0.064	0.113^*^	0.109^*^	0.061	−0.018	−0.044	−0.081
Number of employees	0.099	0.045	0.032	0.008	−0.034	0.183^***^	0.165^**^	0.169^**^
Corporate tenure	0.122^*^	0.051	0.227^***^	0.197^***^	0.160^**^	0.188^***^	0.126^*^	0.112^*^
**Independent variable**
EL	0.821^***^	−0.089	0.794^***^	0.591^***^	0.458	0.866^***^	0.609^***^	−0.229
**Mediating variable**
TKS				0.247^***^	0.146^**^		0.106^*^	0.046
JE							0.214^***^	0.177^**^
**Moderating variable**
CGO		−0.195			0.315			−0.665^**^
**Interaction**
EL × CGO		0.117^*^			−0.005			0.160^**^
*R* ^2^	0.324	0.427	0.405	0.446	0.489	0.425	0.469	0.495
*F-*value	29.484^***^	34.167^***^	41.854^***^	42.366^***^	38.929^***^	45.496^***^	40.591^***^	35.771^***^
Δ*R*^2^	0.324	0.103	0.405	0.041	0.043	0.425	0.044	0.026
*ΔF*	29.484^***^	32.917^***^	41.854^***^	27.443^***^	15.342^***^	45.496^***^	15.299^***^	9.219^***^

*n = 376*;

*** *p < 0.001*,

** *p < 0.01*,

* *p < 0.05*.

Table 3 shows that there was a significant positive association of EL on both TKS and JE (M1, β = 0.821, *p* < 0.001; M3, β = 0.794, *p* < 0.001). When both independent (EL) and mediating variables (TKS and JE) were added to the analysis of EP, both EL (M7, β = 0.609, *p* < 0.001), TKS (M7, β = 0.106, *p* < 0.05) and JE (M7, β = 0. 214, *p* < 0.001) showed a significant positive association on EP. Hayes' (2013) SPSS PROCESS Macro (model 6) was used to obtain a preliminary idea of the direct vs. indirect associations in the model. Table 4 again validates hypothesis 1 and points to the presence of a mediating association of EL in affecting EP [*b* = 0.257, *SE* = 0.058, *95% CI* = (0.144, 0.372), excluding 0].

**Table 4:** Mediating effect.

	***b***	***SE***	**Bootstrap *95% CI***	**Ratio of indirect to total effect**
Total effect	0.866	0.065	(0.739, 0.994)	
Direct effect	0.609	0.079	(0.455, 0.764)	70.323%
Indirect effect	0.257	0.058	(0.144, 0.372)	29.677%

*b, unstandardized regression coefficients; SE, standard errors; CI, confidence intervals*.

**Figure 3:**
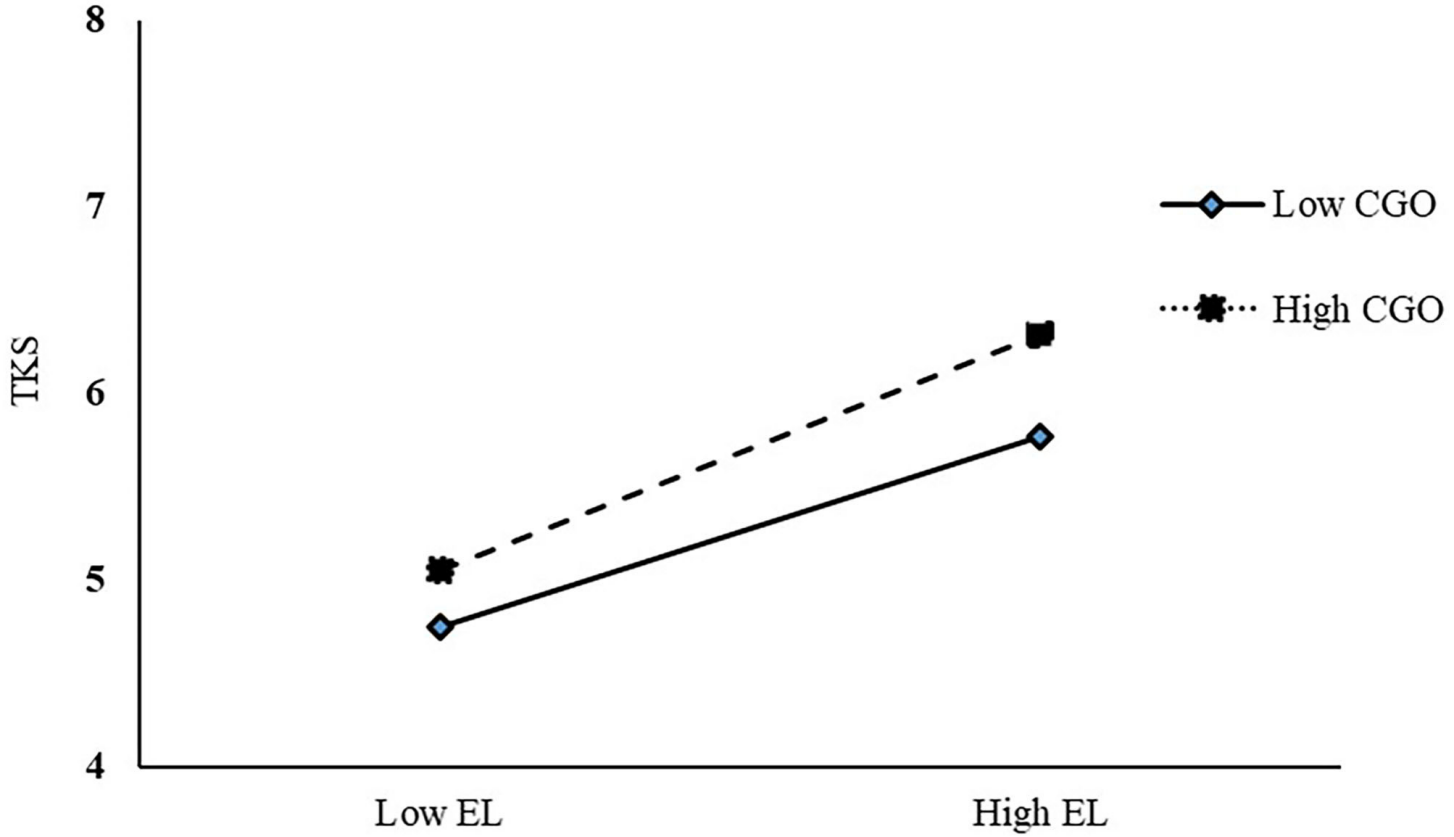
Analysis of moderating effect.

Model 83 was used in SPSS PROCESS Macro (Hayes, 2013) to test the mediating (H2, H3, and H4) and moderating hypotheses (H5, H6, and H7). The effect of EL on TKS was observed under two different CGO according to the procedure recommended by Cohen et al. (2014). The positive effect observed between EL and TKS was more significant when employees were in a high CGO environment (Figure 3). Therefore, hypothesis 5 was supported.

Table 5 shows the mediating association of TKS and JE separately, and the serial mediating association of TKS and JE. The mediating association of TKS between EL and EP was low [*b* = 0.050, *SE* = 0.026, *95% CI* = (0.004, 0.104)], medium [*b* = 0.069, *SE* = 0.031, *95% CI* = (0.008, 0.131)] and high [*b* = 0.087, *SE* = 0.040, *95% CI* = (0.010, 0.168)] (Table 5). The mediating association of JE between EL and EP was significant with a 95% bootstrap confidence interval of 0.088 to 0.269 [*b* = 0.172, *SE* = 0.046, *95% CI* = (0.088, 0.269)]. The serial mediating association of TKS and JE between EL and EP was low [*b* = 0.025, *SE* = 0.011, *95% CI* = (0.007, 0.049)], medium [*b* = 0.034, *SE* = 0.012, *95% CI* = (0.014, 0.060)] and high [*b* = 0.043, *SE* = 0.016, *95% CI* = (0.017, 0.079)]. None of these confidence intervals include 0, so hypothesis 2, hypothesis 3, and hypothesis 4 were confirmed. Furthermore, Table 5 shows that the mediating association of TKS and the serial mediating association of TKS and JE were strengthened with the elevation of CGO. Therefore, hypothesis 6 and hypothesis 7 are supported.

**Table 5:** Conditional process analysis.

**Mediator**	**Path**	**Conditional indirect effects of CGO**			
		**Condition**	***b***	***SE***	**Bootstrap *95% CI***
TKS and JE	EL → TKS → JE → EP	Low	0.025	0.011	(0.007, 0.049)
		Middle	0.034	0.012	(0.014, 0.060)
		High	0.043	0.016	(0.017, 0.079)
TKS	EL → TKS → EP	Low	0.050	0.026	(0.004, 0.104)
		Middle	0.069	0.031	(0.008, 0.131)
		High	0.087	0.040	(0.010, 0.168)
JE	EL → JE → EP	None	0.172	0.046	(0.088, 0.269)

*b, unstandardized regression coefficients; SE, standard errors; CI, confidence intervals*.

## Discussion

In this study, participants expressed positive attitudes toward EL. It was confirmed that EL could improve EP and the impact pathways between EL and EP were explored. This study examined the mediating role of TKS between EL and EP. EL actively encourages employees to seize opportunities and promote knowledge sharing practices (Riana et al., 2020). EL is enthusiastic and energetic, and employees indicated that EL could facilitate their TKS with each other. TKS creates an inclusive and open organizational climate, which benefits EP in start-ups. Knowledge sharing as an intangible resource contributes significantly to innovation (Fernandes Crespo et al., 2021). This study also confirmed the mediating role of JE. Subjects acknowledged that EL could improve their JE, which then improved EP. Leaders can enhance employees' JE by increasing their compatibility with the organization, encouraging informal ties, etc. (Al-Ghazali, 2020). EL stimulates creatively oriented behaviors and utilizes and exploits employees' creative potential (Gupta et al., 2004; Cai et al., 2019). EL can enhance employees' JE sufficiently to retain them and create value, i.e., it can enhance EP.

This study also confirmed the multiple mediating role of TKS and JE. EL gives employees the freedom to explore knowledge to maximize their innovativeness (Riana et al., 2020). EL mobilizes employee initiative (Guberina and Wang, 2021) and enhances EP. TKS allows employees to gain skills and expertise, strengthens the connections and communication between employees, and leads to a greater connectedness to work and higher JE (Karatepe, 2013). Based on employees' responses, it was found that EL stimulate employees' willingness to share tacit knowledge, leading to employees' embeddedness in the organization, and finally to better EP. It verifies the idea that “the stronger one's connection to the job, the more embedded one is in the job,” which ultimately leads to superior EP (Holtom et al., 2006).

Subjects conveyed their preference for CGO. In the context of high CGO, the positive promotion effect of EL on TKS was more obvious, and the serial mediating effect of TKS and JE was enhanced. One of the ways that organizations meet the career needs of their employees is by providing them with CGO (Weng and McElroy, 2012). CGO motivate positive work attitudes and behaviors. Employees feel obligated to show greater commitment to the organization under CGO (Bashir et al., 2020). In this study, employees' positive behavioral and psychological manifestations are TKS and JE, which ultimately shows that employees earn good EP for their organizations. Previous research has noted the moderating effect of perceived CGO as a contextual characteristic (Lu et al., 2016). When EL provides employees with rich growth spaces, their intrinsic emotions are stimulated and “feedback” to the organization is triggered (Weer and Greenhaus, 2020). Knowledge, information, talent, corporate resources, and organizational capabilities are like a box of puzzles waiting to be put together, and strategic leaders can “put the pieces in motion.” The leader's ability to be fully effective and present the “perfect look” is influenced by their ability to assemble the necessary resources. This preservation or enhancement of resources is enhanced with the inclusion of a CGO. The mediating mechanism is more evident with the support of CGO. This proves that CGO is critical in improving organizational growth and performance.

### Theoretical Implications

Many current discussions on authentic leadership (Edú-Valsania et al., 2016) and ethical leadership (Bavik et al., 2018) have taken place, while there have been insufficient discussions of EL. There is still a lack of research analyzing the effects of EL on EP, but this paper starts to fill this gap. First, this study establishes a moderated serial mediation model of EL and EP. This study also validates the mechanism by which EL influences EP from multiple paths, remedying the fact that the extant literature has mostly examined the influence of leadership style on EP from a single level, and provides empirical support for the improvement of EP in start-ups. The initial exploration of EL as an example in Chinese start-ups should complement and extend to the current EP field. These findings broaden the range of predictors of EP and suggest the significance of leadership behavior on employee behavior and psychology. Second, the study highlights the role of TKS. Knowledge sharing is critical to high performance environments (Riana et al., 2020). Third, it expands the research on JE. Most of the previous studies on JE have focused on intention, while ignoring the critical role of JE in analyzing employee job outcomes (Lee et al., 2004; Holtom et al., 2006). Fourth, in analyzing the relationship between EL and EP, this study introduces CGO as a moderating variable in the first stage based on SET, which is a significant innovation. This study clarifies the theoretical model of boundary conditions by using CGO as a moderating variable for the entire research framework. It also enhances the contextual features of the study (Lu et al., 2016). Finally, in the extant literature on EL, case studies have been common (Wang et al., 2012). This paper is a quantitative study, which improves the persuasiveness of the causal relationship between the variables of interest.

### Managerial Implications

This study points out the importance of EL for start-ups. A creative atmosphere is a work environment that should be cultivated (Edú-Valsania et al., 2016), and EL provides this environment for companies. EL can encourage employees to do more useful things for the organization and make them more trusting in the organization, more willing to perform TKS, and more dependent on the work and the company. Managers should exercise their ability to actively promote TKS and integrate knowledge management goals into corporate strategic objectives to improve organizational effectiveness (Castellani et al., 2021) and corporate performance. Firms need to provide employees with opportunities to build and protect job resources, such as employment security, social support, and career development opportunities, to increase their JE and subjective well-being (Ampofo et al., 2017). Employees who have a high sense of belonging to their jobs make more significant contributions to the organization. Business leaders need to focus on non-material incentives to keep employees active. This study points out the importance of CGO, which stimulates positive psychological effects for employees such as increased self-confidence and optimism, and increases job satisfaction (Kahn, 1990). This can allow companies to motivate their employees to hold positive work attitudes, creating an appropriate organizational climate and strong team cohesion.

### Limitations and Directions for Future Research

Although this study contributes significantly to the study of EL, it still has the following shortcomings. First, this study is a cross-sectional study, which cannot determine causality. A longitudinal study should be designed to explore this. Second, the subjects of this study were all employees, and leaders could be included as participants in future research. Third, although this study adopted a series of methods to weaken the homoscedasticity bias of the questionnaire, it still cannot be completely avoided. Fourth, other relevant variables may be selected as moderating variables in future research. Finally, this study only discusses the factors that play a positive role in EL's influence on EP, and subsequent studies may choose to discuss the negative factors affecting this mechanism.

## Conclusion

In this study, a total of 376 start-up employees were selected as a sample to demonstrate the mediating role of TKS and JE between EL and EP, and the moderating role of CGO. Specifically, this study draws the following conclusions: (1) EL has a positive association with EP. (2) TKS and JE can mediate EL and EP, and TKS and JE both play a serial mediating role. (3) CGO is a moderator of the positive association between EL and TKS, and can moderate the mediating effect of TKS on EL and EP and the serial mediating association of TKS and JE on EL and EP. In summary, based on the resource-based view and SET theory, this study explored the mediating role of TKS and JE and the moderating role of CGO. The mechanism of EL's influence on EP in the Chinese context is comprehensively analyzed. Start-ups should actively encourage employees to share tacit knowledge, embed employees in the organization, and provide sufficient CGO. This study enriches the literature on EL and hopes to stimulate scholars' thinking.

## Data Availability Statement

The original contributions presented in the study are included in the article/supplementary material, further inquiries can be directed to the corresponding author.

## Ethics Statement

Ethical review and approval was not required for the study on human participants in accordance with the local legislation and institutional requirements. Written informed consent was obtained from the individual(s) for the publication of any potentially identifiable images or data included in this article. The patients/participants provided their written informed consent to participate in this study.

## Author Contributions

BP and SJ: conceptualization, methodology, formal analysis, and writing—original draft preparation. BP: investigation and funding acquisition. BP and WS: writing—review and editing. BP and ZT: supervision. All authors have read and agreed to the published version of the manuscript.

## Funding

This research was supported by the National Natural Science Foundation of China, Grant Number 71804119 and China Postdoctoral Science Foundation, Grant Number 2019M663482.

## Conflict of Interest

The authors declare that the research was conducted in the absence of any commercial or financial relationships that could be construed as a potential conflict of interest.

## Publisher's Note

All claims expressed in this article are solely those of the authors and do not necessarily represent those of their affiliated organizations, or those of the publisher, the editors and the reviewers. Any product that may be evaluated in this article, or claim that may be made by its manufacturer, is not guaranteed or endorsed by the publisher.
